# Treatment of Threatened Abortion by Hypodermic Injections of Morphine

**Published:** 1873-02

**Authors:** A. B. Isham

**Affiliations:** Cincinnati, Ohio


### SELECTIONS.

### TREATMENT OF THREATENED ABORTION BY HYPODERMIC INJECTIONS OF MORPHINE.

BY A. B. ISHAM, M.D., OF CINCINNATI, OHIO.

It is the design of this paper to treat the subject of threatened miscarriage from a clinical stand-point, where the hazard to the mother is remote, while the life of the fœtus is in imminent danger. All questions, then, not relating to the preservation of the offspring, are set aside.

Upon the question of preventing abortion after the occurrence of well-marked bearing-down pains, dilation of the os, or hemorrhage, authorities vary somewhat, though the weight of authority is indeed adverse to the chances of preserving the fœtus under the condition considered; yet while many authors admit that our efforts in this direction may sometimes be crowned with success, it is the universal verdict that such result is exceptional. The only conditions generally acknowledged as hopeless are fetid discharges, and partial protrusion of the fœtus or its membranes through the os. Even in the former event, if the prospects be not favorable for a speedy expulsion of the womb contents, we may put a stop to uterine efforts, and indulge a very slight hope that fœtal life may not be destroyed; for there is a possibility that the fetor may be due to other causes than the decomposition of the ovum. Upon this point Cazeaux well observes:—*

"For after all, whenever death of the fœtus must have been either the cause or effect of the primary symptoms, what do we risk in calming or arresting the uterine contraction? because, as we have already seen, the dead child may remain long within the intact membranes without any unfavorable consequences resulting to the mother. And besides, as it is impossible to ascertain its death with any degree of certainty prior to the fifth month of gestation, we must act in such doubtful cases just as if it were living."

*Theoretical and Practical Obstetric Med., 5th Am. ed., pp. 582–3.

I have preserved a record of seven cases of menaced abortion, in all of which there existed forcible uterine contractions, hemorrhage and dilatation of the os. In every instance the uterine contractions and hemorrhage were arrested and the os contracted from the administration of morphia, by the mouth and hypodermically, in full doses and at short intervals. In four cases, the patients were carried to full term, and voided fully developed children. In the three unsuccessful cases, expulsion occurred in from three days to two weeks. In two of these cases, there was found fetor of the discharges on first examination, so that they may bé excluded from the count, as almost necessarily hopeless. Of the remaining cases four out of the five were successful, and of the four, three were treated by hypodermic injections of morphia. These cases I submit in detail.

Case I.—G., colored, æt. 37: five months advanced in pregnancy. December 30, 1869, was kicked and struck in the abdomen by a man. Vomiting; severe and very frequent bearing-down pains; os uteri soft and dilated an inch and a quarter; rather free hemorrhage. Administered a hypodermic injection of $\frac{1}{4}$ gr. morphia, in twenty minutes another of like quantity, and in twenty minutes more the third, when the pains ceased and the patient fell asleep. Saw no more of her, but learned that she went to full term and was delivered by a midwife of a healthy, full-grown child.

Care II.—M. C., mulatto, æt. 32; mother of four children; four and a half months advanced in pregnancy. Previous to this pregnancy, had had three miscarriages in succession at about four months. Saw her the morning of May 27, 1870. Stomach very irritable; vomiting and retching. Uterine contractions sharp and regular; os soft and dilated, the size of a half dollar; slight hemorrhage. Gave her two injections of $\frac{1}{4}$ gr. of morphia each, which suspended the pains. In the afternoon pains returned, when she was given two more injections with the same result as before. Uterus took on expulsive action again in the evening, when three hypodermic injections of $\frac{1}{4}$ gr. each were necessary to insure quiet.

28th.—Made three visits, each time bringing the injections into requisition to govern uterine contraction.

29th.—One visit, with two injections, sufficed.

31st.—As the irritability of the stomach had subsided, and the pains were not severe, gave two morphia powders of $\frac{1}{4}$ gr. each by the mouth; after which no more trouble was experienced, and the woman was delivered at full term, October 16, 1870, of a healthy, well-developed female infant.

*Remarks.*—Three miscarriages in succession had occurred to this woman, doubtless produced by her own agency. Her discouraged husband was seriously resolved "to quit breedin' with her, 'cause she would drap 'em, spite of everything." She was averse to treatment in this instance, declaring that she did not want children, and, when not under the influence of morphia, notwithstanding repeated injunctions to keep quiet, she would persist in exercising herself.

Case III.—Mrs. C., German, æt. 34; mother of two living children and two still-born. Called to her April 26, 1871. According to her data there lacked about two weeks of full term. She was suffering severe bearing-down pains, accompanied with gushes of blood in considerable quantity. Os rigid and dilated just sufficient to pass the index finger. Could feel just through the os a thick pulpy substance, which was decided to be the placenta. Administered $\frac{1}{3}$ gr. morphia by hypodermic injection, which relived the pains and stopped the hemorrhage. She was delivered May 3d, of a full-grown still-born male child, by turning, with the aid of Dr. Keyt.

*Remarks.*—In the absence of any forcible uterine contractions the processes of dilatation and delivery were entirely manual. The placenta was implanted half way over the os, and the child perished from hemorrhage, for the fœtal heart-sounds could be detected nearly up to the time of turning. In the first hemorrhage on the 26th of April, the life of the fœtus was undoubtedly saved by the prompt exhibition of the morphia injection, and but for the uterine inertia, the chances of its being born alive had not been altogether unfavorable.

My cases are only three, it is true. But, as in all the same treatment obtained, they may serve as good a purpose for comparison as a much larger number. Particularly so, as they were all of that class in which the highest obstetrical authority would expect failure; and when, from the employment of other means, in cases marked by symptoms very much milder, I have not a few times experienced failure myself.

All the advocates' of opium, with the exception of Tyler Smith, Byford and Tanner, advise its administration per rectum. This is probably and likely because the irritable stomach so common in cases of threatened miscarriage may not tolerate so nauseous a medicine as opium. In the manner of administration may have been the small measure of success. It is well known that medicaments introduced into the rectum act in a very uncertain manner; and it will be readily seen how opium or its alkaloids, given per orem to a patient vomiting and retching, might fail to avert a miscarriage. Just where these means have failed and will fail again, is where the hypodermic injections would prove invaluable.

It is useless to enjoin quiet. The patient is not like likely to be quiet, suffering the pains incident to uterine expulsive efforts, her mind perturbed by fears for her own welfare and that of her child. Too often, too, the fruit of conception is regarded as an incumbrance, which it would be happiness to lose, and constant activity is one of the most popular recipes to the attainment of such end. Cold applications, leeching and blood-letting, would prove in most instances a waste of precious time; besides at this day such practice would be esteemed a barbarity, and not without some reason.

The prime elements of danger to the life of the fœtus are, *first*, muscular contractions of the uterus, and *second*, hemorrhage. The latter most commonly results from the operation of the former; but it matters nothing as to the many and various causes tending to produce these phenomena. Considerations of safety imperatively demand a speedy arrest of uterine muscular action and hemorrhage, securing which, we may go about investigating and correcting the cause with comparative leisure.

The use of morphia by hypodermic injection is the most speedy and certain means we possess of effecting such desiderata. It will do even more. Its influence spreads over the brain, and at the same time that it suspends contraction of the uterine muscular fibres, it insures quiet and rest to the whole system, even against the patient's will. It is applicable to any case of threatened abortion, and I believe that its general use would render success at least equal, if not the rule, instead of the exception, as now announced by obstetricians. Of course I am aware that there are occasional individuals who do not well bear the exhibition of opium in any form, but I hold with Byford: "I do not forget, in thus speaking freely about the use of opium, that peculiarities in some persons make it almost useless; yet I remember the utter unreliability of other remedies in these cases, and risk such disagreement.—*American Journal of Medical Sciences.*

